# The Role of EUS in Advanced Endoscopic Procedures and Therapeutics—Advancing the Field to Greater Heights

**DOI:** 10.3390/jcm12144557

**Published:** 2023-07-08

**Authors:** Rupinder Mann, Hemant Goyal, Abhilash Perisetti

**Affiliations:** 1 Department of Gastroenterology, University of Arkansas for Medical Sciences, Little Rock, AR 72205, USA; 2 Instructor, Department of Surgery, Division of Endoluminal Surgery & Interventional Gastroenterology, University of Texas Health Science Center, Houston, TX 77030, USA; 3 Department of Gastroenterology and Hepatology, Kansas City VA Medical Center, 4801 Linwood Blvd, Kansas City, MO 64128, USA

Endoscopic ultrasound (EUS) provides high-resolution and real-time visualization of various layers of the gastrointestinal (GI) tract and beyond by combining ultrasound technology with endoscopic visualization. The EUS was first developed in the 1980s. Over the last few decades, the EUS expanded from a mere diagnostic tool to an advanced therapeutic intervention tool [1]. The GI tract provides a unique opportunity to access other structures in the mediastinum and abdomen. The extension of the EUS role from diagnostic to therapeutic tool opened the doors for various pancreaticobiliary interventions in cases with failed endoscopic retrograde pancreatography (ERCP) and in patients with surgical alerted anatomy where traditional ERCP is difficult. In patients with obstructed main PD, where ERCP failed or alerted surgical anatomy, making it challenging to access the PD by ERCP, EUS-guided PDD with either EUS-guided rendezvous or transmural drainage techniques provided an alternative to the surgical management, which has higher mortality and morbidity [2]. Teh JL et al. discussed various techniques and outcomes of EUS-guided pancreatic duct drainage (PDD). In this article, the authors detailed multiple tools for the interventional endoscopist to obtain access to PD when traditional approaches fail [2].

EUS-guided fine needle aspiration or biopsy was shown to be valuable in diagnosing and managing pancreatic neuroendocrine tumors (PNET) by improving the grading of tumors and the detection of malignancy at an earlier stage. Pancreatic cancers have high morbidity and mortality. One rare form of pancreatic tumor is the pancreatic neuroendocrine neoplasm (PNET). PNETs are slow-growing tumors, but they carry a poor prognosis if they metastasize to other areas. The mainstay treatment for large, symptomatic, and metastatic pancreatic neuroendocrine tumors was pancreatic surgery. Paik WH et al. [3] discussed the controversies in the management of PNET, including the advancement of the therapeutic utility of EUS in these conditions. EUS-guided treatment options could be offered in these patients, like ethanol cauterization or radiofrequency ablation as an alternative to surgery that carries a high risk of morbidity and mortality, especially in high-risk patients who are poor surgical candidates [3].

Similar to pancreatic duct drainage, biliary duct access could be challenging in selected patients. In recent years, EUS-guided BD was used as an alternative to percutaneous transhepatic biliary drainage in cases of biliary obstruction where ERCP failed to access the bile duct. EUS-guided BD is carried out either by rendezvous with ERCP or antegrade stenting and bilioenterostomy. EUS-guided hepaticogastrostomy (EUS-HG) is one of the most frequently performed EUS-guided BD, as it is preferred in patients with duodenal stenosis, surgically altered anatomy, and high-grade hilar stenosis [4]. This procedure involves accessing the biliary system from the stomach or jejunum (in case of post-gastrectomy), guidewire passage, track dilation, and placement of either a self-expandable metallic stent or plastic stent. This procedure is technically challenging, with adverse events like stent migration and bile leaks [4]. These adverse events of the biliary leak are more frequent with plastic stents than metal stents. A multicenter retrospective study conducted by Kobori I et al. showed a reduction in bile leakage with a plastic stent when track dilation and bile aspiration were carried out with an ERCP contrast catheter [5]. In patients with acute cholecystitis who were not surgical candidates, endoscopic transpapillary gallbladder drainage (ETGBD) provided an alternative option to percutaneous transhepatic gallbladder drainage. 

The role of EUS was extended to access gallbladder (GB) with a high success rate. Traditionally, gallbladder drainage is performed percutaneously or recently with EUS-guided GB drainage (using lumen apposing metal stents). However, in patients with massive ascites or inaccessible anatomical locations, these methods may not be possible. In these cases, use of an endoscopic approach to access GB can be performed via a transpapillary approach (through the sphincter of Oddi). A duodenoscope is used to access the biliary tree and reach GB via the cystic duct. In individuals where the cystic duct cannot be located, EUS can offer visualization of GB, including the cystic duct, to gain access. Maruta A et al. [6], in a multicentric study, evaluated a novel method of nasogallbladder drainage (via transpapillary route distally and nasal tube proximally) by cutting the tube in the stomach for the GB contents to drain into the stomach. The authors concluded that endoscopic nasogallbladder drainage by cutting the tube in the stomach could be a safe alternative for acute cholecystitis who cannot tolerate traditional methods [6]. 

EUS was used to access mediastinal structures, linitis plastica, and anal fissure management. EUS-guided fine needle aspiration (FNA) provides a minimally invasive option for diagnosis of mediastinal masses, especially in the posterior mediastinum where EBUS is not possible. A retrospective analysis by Assisi D et al. showed a high accuracy of mediastinal mass diagnosis by EUS-guided FNA with an overall sensitivity of 85% and negative predictive value of 56%, and accuracy of 87.5%, thus reducing the need for surgical procedure for various cancer staging [7]. EUS emerged as a novel technique over the past decade for the diagnosis and staging of GI cancer, especially in infiltrative GI cancer, by allowing deeper layers biopsy. Gastric linitis plastica is the type of gastric cancer that arises from submucosa and, thus, can be easily missed with standard endoscopic biopsy. It carries a poor prognosis partly due to diagnosis at an advanced stage, given the low yield on endoscopic biopsies. EUS-guided fine needle aspiration biopsy provides a minimally invasive diagnostic modality compared to other more invasive options for diagnosing patients with gastric linitis plastica when standard endoscopic biopsy fails [8]. The advent of various EUS-guided therapeutic interventions also improved the management of chronic anal fissures, especially in cases where medical treatment failed. EUS-guided botulinum toxin injection into the internal anal sphincter showed promising results in these patients with chronic anal fissures, as EUS provides direct visualization, thus ensuring injection to the internal anal sphincter only. However, with conventional endoscopic botulinum toxin injections in patients with anal fissures, injections are administered blindly without direct visualization, so they can be injected into the submucosa or muscle, thus decreasing the efficacy of treatment [9].

The diagnostic and therapeutic capabilities of EUS will continue to advance, especially in combination with AI technology. Various artificial intelligence (AI) models can assist in the detection and characterization of lesions including subepithelial lesions in combination with EUS [10]. It potentially could also improve the EUS training by improving recognition of anatomic structures [11]. Although still in the early stages, AI-EUS models [12] are showing promising results in detecting pancreatic cancer early and differentiating it from other clinically relevant pancreatic abnormalities like chronic pancreatitis and autoimmune pancreatitis.
